# Seroprevalence of Hepatitis B Surface Antigen, Antibodies to the Hepatitis C Virus, and Human Immunodeficiency Virus in a Hospital-Based Population in Jaipur, Rajasthan

**DOI:** 10.4103/0970-0218.62588

**Published:** 2010-01

**Authors:** Smita Sood, Shirish Malvankar

**Affiliations:** Department of Laboratory Medicine, Fortis Escorts Hospital, Jaipur and Super Religare Laboratories (Formerly SRL Ranbaxy) Reference Lab, Mumbai, India

**Keywords:** Hepatitis B, hepatitis C, HIV, Rajasthan, seroprevalence

## Abstract

**Background:**

Hepatitis B, hepatitis C, and HIV infections are a serious global and public health problem. To assess the magnitude and dynamics of disease transmission and for its prevention and control, the study of its seroprevalence is important. A private hospital catering to the needs of a large population represents an important center for serological surveys. Available data, at Rajasthan state level, on the seroprevalence of these bloodborne pathogens is also very limited.

**Objective:**

A study was undertaken to estimate the seroprevalence of hepatitis B surface antigen (HBsAg) and antibodies to hepatitis C (anti-HCV Ab) and human immunodeficiency virus (anti-HIV Ab) in both the sexes and different age groups in a hospital-based population in Jaipur, Rajasthan.

**Materials and Methods:**

Serum samples collected over a period of 14 months from patients attending OPDs and admitted to various IPDs of Fortis Escorts Hospital, Jaipur, were subjected within the hospital-based lab for the detection of HBsAg and anti-HCV Ab and anti-HIV Ab using rapid card tests. This was followed by further confirmation of all reactive samples by a microparticle enzyme immunoassay (Abbott AxSYM) at Super Religare Laboratories (formerly SRL Ranbaxy) Reference Lab, Mumbai.

**Results:**

The seroprevalence of HBsAg was found to be 0.87%, of anti-HCV Ab as 0.28%, and of anti-HIV Ab as 0.35%.

**Conclusion:**

The study throws light on the magnitude of viral transmission in the community in the state of Rajasthan and provides a reference for future studies.

## Introduction

Hepatitis B and C infections are a serious global and public health problem. Hepatitis B virus (HBV) is highly infectious and can be transmitted covertly by percutaneous routes and overtly by blood transfusion. The hepatitis B surface antigen (HBsAg) in serum is the first seromarker to indicate active HBV infection, either acute or chronic.(1) Worldwide over 2 billion people have been infected with HBV and more than 350 million have chronic HBV infection.(2) India has been placed into the intermediate zone of prevalence of hepatitis B (2–7% prevalence rates by WHO).(3) This infection is a leading cause of morbidity and mortality, not only because of the acute illness but also due to its chronic sequelae like chronic hepatitis, cirrhosis, and hepatocellular carcinoma, accounting for more than a million deaths worldwide.(4) An effective vaccine is available for over two decades and has brought about remarkable changes in the global epidemiology of HBV infection.

Among the viral hepatitis strains, hepatitis C virus (HCV) is especially dangerous in that its morbidity rate is high as it establishes a state of chronic infection in as many as 85% of acutely infected patients, whereas about 15% of acutely infected patients spontaneously clear the infection.(5) Chronic hepatitis C is a ubiquitous disease affecting around 200 million people worldwide.(6) The major channels of HCV transmission are all related to exposure to blood and blood products. The presence of anti-hepatitis C virus antibody (anti-HCV Ab) indicates previous exposure to hepatitis C virus. This antibody is present in only 40% of acute infections but in more than 95% of chronic infections.(7) In India, antibodies against HCV are present in approximately 15 million people with a prevalence rate of 2%.(8)

The HIV/AIDS epidemic is one of the largest public health crises of the 21st century. While the epidemic has spread over the past two decades, a cure or vaccine for HIV remains elusive. The HIV prevalence estimates have come under increased scrutiny in recent years and with the availability of more reliable data, the estimated number of HIV-infected people in India has been revised downward from 5.7 million to 2.5 million in 2007.(9) In India, the predominant mode of HIV transmission is through heterosexual contact.

To understand and assess the magnitude and dynamics of transmission of a disease in a community and for its control and prevention, the assessment and study of its prevalence is very important. Community-based seroprevalence studies are difficult to conduct in a developing country because of socioeconomic hurdles and logistic difficulties. India has a strong private health care system that caters to more than one-half ambulatory and two-thirds outpatient care.(10) As a result, a large amount of clinical information is available in a private health care setting. A private hospital catering to the needs of a large population thus represents an important center for serological surveys. Also, the available data at Rajasthan state level on the seroprevalence and distribution of these bloodborne pathogens is limited.

It was against the above backdrop that the present study was undertaken to estimate the seroprevalence of HBs Ag and antibodies to hepatitis C and HIV in both the sexes and different age groups in a hospital-based population in Jaipur, Rajasthan.

## Materials and Methods

### Setting

This study was carried out in the Serology Section of the Department of Laboratory Medicine, Fortis Escorts Hospital, Jaipur, Rajasthan, after an approval from the institutional review committee. HIV antibody detection was performed only after pretest counseling and informed consent of the patient. Reactive results of HIV antibody testing were disclosed only after posttest counseling.

### Patients and period of the study

Patients who registered at the OPDs or were admitted to the IPDs of this private hospital and were advised to undergo HIV and HCV antibody testing and hepatitis B screening were included in the study. The study extended over a period of 14 months from July 2007 to August 2008.

### Specimen

A 5-ml venous blood sample was collected in from all patients who came with lab requisitions for the testing of HBsAg and HIV and HCV antibodies. The blood was allowed to clot for 45 min at room temperature and the serum was separated after centrifugation at a low speed. The serum sample was then subjected to requested tests.

### Serology

The serum was tested for HIV antibodies using a rapid card test – HIV Tridot Rapid HIV 1 and 2 (Biomed Industries). Samples testing reactive with this method were rechecked in-house by two other rapid tests – Determine HIV½ (Abbott Laboratories) and Signal HIV 1/2 (Span Diagnostics). Samples were further confirmed for HIV antibodies at Super Religare Laboratories (formerly SRL Ranbaxy) Reference Lab, Mumbai, by an enzyme immunoassay (MICROLISA – J Mitra and Co.) and by a microparticle enzyme immunoassay (Abbott AxSYM).

IgG antibodies to HCV were determined using a rapid card method – HCV Tridot (Biomed Industries). Samples reactive by this test were rechecked in-house by another rapid test Hep-Alert C (RFCL) and were further confirmed by the microparticle enzyme immunoassay (Abbott AxSYM) at Super Religare Laboratories Reference Lab, Mumbai.

HBsAg was determined using a rapid card method Hepacard (Biomed Industries). Samples reactive with this test were rechecked in-house by Crystal HBsAg Dipstick (Span Diagnostics) and were further confirmed by the microparticle enzyme immunoassay (Abbott AxSYM) at Super Religare Laboratories Reference Lab, Mumbai. All the tests were performed in accordance with the manufacturer's instructions with adequate controls.

### Results

In all, 3196 serum samples were processed for HBsAg detection, 1392 serum samples were tested for hepatitis C antibodies, and 1980 serum samples were tested for HIV antibody detection over the 14-month period. Tables 1–3 show the age and sex distribution of the hospital-based population with hepatitis B, C, and HIV seropositivity, respectively. The seroprevalence of HBsAg was found to be 0.87%, of anti-HCV Ab as 0.28%, and of anti-HIV Ab as 0.35%. The seroprevalence for HBsAg among males and females was 1.04% and 0.58%, respectively. The highest seroprevalence of anti-HCV was found in males above the age of 61 years. The highest seroprevalence for anti-HIV was found in the age group 31-40 years. All the patients testing seropositive for HIV demonstrated HIV-1 antibodies.

**Table 1:** Age and sex distribution of the hospital-based population with hepatitis B seropositivity (n=3196)

Age (years)	No. of males tested	No. of females tested	No. of males with HBsAg detected (%)	No. of females with HBsAg detected (%)	Total HBsAg-positive cases (%)
0–10	18	09	00 (0)	00 (0)	00 (0)
11–20	25	20	02 (8)	00 (0)	02 (4.4)
21–30	238	189	03 (1.2)	01 (0.52)	04 (0.93)
31–40	264	198	01 (0.37)	01 (0.50)	02 (0.43)
41–50	378	196	05 (1.3)	01 (0.51)	06 (1.04)
51–60	525	262	04 (0.76)	01 (0.38)	05 (0.63)
Above 61	556	318	06 (1.07)	03 (0.94)	09 (1.02)
Total	2004	1192	21 (1.04)	07 (0.58)	28 (0.87)

**Table 2:** Age and sex distribution of the hospital-based population with anti-HCV Ab seropositivity (*n* = 1392)

Age (years)	No. of males tested	No. of females tested	No. of males with anti-HCV Ab detected (%)	No. of females with anti-HCV Ab detected (%)	Total anti-HCV Abpositive cases (%)
0–10	04	02	00 (0)	00 (0)	00 (0)
11–20	15	09	00 (0)	00 (0)	00 (0)
21–30	50	30	00 (0)	00 (0)	00 (0)
31–40	120	80	00 (0)	00 (0)	00 (0)
41–50	1128	107	01 (0.78)	01 (0.93)	02 (0.85)
51–60	235	146	00 (0)	00 (0)	00 (0)
Above 61	307	159	02 (0.65)	00 (0)	02 (0.42)
Total	859	533	03 (0.35)	01 (0.18)	04 (0.28)

**Table 3:** Age and sex distribution of the hospital-based population with anti-HIV Ab seropositivity (*n* = 1980)

Age (years)	No. of males tested	No. of females tested	No. of males with anti-HIV Ab detected (%)	No. of females with anti-HIV Ab detected (%)	Total anti-HIV Abpositive cases (%)
0–10	18	12	00 (0)	00 (0)	00 (0)
11–20	31	18	01 (3.2)	00 (0)	01 (2.04)
21–30	109	78	00 (0)	01 (1.2)	01 (0.53)
31–40	168	126	02 (1.1)	02 (1.5)	04 (1.36)
41–50	193	134	00 (0)	00 (0)	00 (0)
51–60	330	194	01 (0.3)	00 (0)	01 (0.19)
Above 61	362	207	00 (0)	00 (0)	00 (0)
Total	1211	769	04 (0.33)	03 (0.39)	07 (0.35)

## Discussion

The seroprevalence of hepatitis B surface antigen of 0.87% was noted in our hospital-based population. Lodha *et al*. (2001) in their review article on hepatitis B epidemiology have suggested the true prevalence rate in India as 1–2%.(11) There is a wide variation in the prevalence in different regions of our country, and the highest prevalence has been reported among the aborigines of Andaman as well as from Arunachal Pradesh.(2)

In a study conducted in a hospital-based population at Kathmandu Medical College Hospital, Nepal, the prevalence rate of viral hepatitis B was found to be 2.5%.(12) The prevalence of HBsAg in patients attending a surgical OPD in Rawalpindi, Pakistan, has been reported as 2.28%.(13) The prevalence of hepatitis B varies from country to country and depends upon a complex mix of behavioral, environmental, and host factors. In general, it is lowest in countries or areas with high standards of living (e.g., Australia, North America, North Europe) and highest in countries or areas with low socioeconomic Levels (e.g., China, South East Asia, South America).

The seroprevalence of hepatitis B among males and females in our study was 1.04% and 0.58%, respectively. In a study on hospitalized patients in Manipal, Dutta *et al.* observed HBsAg positivity of 35.3% in males versus 19.3% in females.(14) No plausible explanation has been given for the higher prevalence in males in the general population but probably females clear the HBV more efficiently as compared to males.(3)

The seroprevalence of HCV among our hospital-based population was found to be 0.28%. This seroprevalence is much lower than the 1.7% seroprevalence reported in an earlier study from Jaipur (Rajasthan) in 2007 by Sharma *et al*.(15) In India, the seroprevalence of HCV varies among hospital-based populations with 1.57% reported from Cuttack (Orissa),(6) 4.8% from Pondicherry,(16) and 2.46% from Jodhpur (Rajasthan).(7)

Geographical variation in the seroprevalence of HCV has also been documented by Sun *et al.*(17) in Taiwan. Hospital-based studies from Mauritius,(18) Ethiopia,(19) and Pakistan(20) have showed a seroprevalence of HCV of 5.9%, 6%, and 9%, respectively.

In our study the four cases reactive for HCV antibodies belonged to age groups 41–50 years and ≥61 years. The small sample size of reactive cases in our study does not allow data to be compared with other reports. However, Ramarokoto *et al.*(21) in their study on the seroprevalence of hepatitis C in urban areas of Madagascar reported that the prevalence did not differ significantly according to gender but it increased with age.

The seroprevalence of antibodies to HIV in our hospital population was 0.35%. This is in accordance with the 2006 estimates of NACO (National AIDS Control Organization), NIHWF (National Institute of Health and Family Welfare), and NMS (National Medical Statistics) which suggest that the national adult HIV prevalence in India is 0.36%.(22) Since the first report of HIV infection in India in 1986, the virus has spread all over the country though there is geographical variation.(23) The prevalence of HIV in the low-risk group of hospital patients comprising medical, surgical patients and antenatal cases at the University Hospital of BHU, Varanasi, has been reported as 0.37%.(24)

The seroprevalence of HIV antibodies among 183,912 persons screened in a teaching tertiary care hospital in Haryana was reported to be 0.64%.(25) In another study on HIV seroprevalence in a hospital-based population in Hyderabad (Andhra Pradesh), 04.3% males and 2% females tested positive for HIV and the highest seroprevalence was reported in males and females of age group 21–30 years.(26) However, the highest seroprevalence for anti-HIV Ab in our study was found in age group 31–40 years. All the patients testing seropositive for HIV Ab in our study demonstrated HIV-1 antibodies. Both HIV serotypes 1 and 2 exist in India but HIV-1C is the commonest subtype reported.(23)

The estimation of the seroprevalence of HIV provides essential information for an effective implementation of AIDS control program and also for monitoring HIV spread within the country.

This is the first report defining rates of infection with all these bloodborne agents among the hospital-based population in Jaipur, Rajasthan. The observed rates likely reflect the patient population served by our hospital and do not necessarily apply to other centers. However, the study does throw light on the dynamics of viral transmission in the community in this part of the country and provides a good reference for future studies because of the large number of cases investigated.
